# EEG-based monitoring of mental fatigue during virtual-reality motor imagery tasks

**DOI:** 10.3389/fnbeh.2026.1810723

**Published:** 2026-05-21

**Authors:** Nina Evetović, Roman Rosipal, Arina Polyanskaya, Zuzana Rošťáková, Katarina Dvornák, Martin Vankó, Štefan Korečko, Leonardo Jose Trejo

**Affiliations:** 1 Institute of Measurement Science (IMS), Slovak Academy of Sciences (SAS), Bratislava, Slovakia; 2 Pacific Development and Technology, LLC, Capitola, CA, United States; 3 Middle European Interdisciplinary Master's Programme in Cognitive Science, University of Vienna, Vienna, Austria; 4 Department of Computers and Informatics, Faculty of Electrical Engineering and Informatics, Technical University of Košice, Košice, Slovakia

**Keywords:** brain-computer interface, electroencephalography, mental fatigue, motor imagery, neurorehabilitation, N-way Partial Least Squares (N-PLS), virtual reality

## Abstract

Prolonged motor-imagery training in immersive virtual-reality environments can induce mental fatigue, reducing engagement and potentially limiting the effectiveness of neurorehabilitation. This study investigated neural markers of mental fatigue by recording electroencephalography (EEG) from healthy participants during extended motor-imagery and control sessions in a head-mounted display setup. Multidimensional analysis was applied to extract spectral, spatial, and temporal features while using a novel deflation step for removing task-related motor components to isolate fatigue-specific activity. Evidence of mental fatigue was consistently seen in parieto-occipital alpha-band modulation, with increases in alpha power corresponding to subjective reports and EEG-based measures of mental fatigue. The derived models were robust to common EEG artifacts and demonstrated consistent fatigue estimation across tasks and sessions. These findings suggest that individualized neural markers can enable real-time monitoring of fatigue (with an accuracy of 83.49 ± 6.34%), allowing adaptive adjustments of task difficulty or pacing in brain–computer interface systems. This work advances understanding of the neurophysiological dynamics of mental fatigue during immersive motor-imagery tasks and provides a foundation for designing more effective, personalized neurorehabilitation protocols.

## 1 Introduction

Mental fatigue is a cognitive state characterized by a decline in cognitive and behavioral performance, manifesting as impaired attention, reduced capacity for planning, and diminished ability to adapt strategies in response to challenges or negative outcomes (Boksem et al., 2005). It typically arises from prolonged cognitive, perceptual, or sensorimotor demands, especially in tasks requiring sustained attention, complex decision-making, or continuous mental effort (Kunasegaran et al., 2023; Talukdar et al., 2019). The development of mental fatigue is also influenced by task characteristics, particularly cognitive load and the level of cognitive control required (Tanaka et al., 2012).

Mental fatigue can be assessed using multiple metrics, including behavioral measures (reaction time and task performance accuracy) (Boksem et al., 2005; Ackerman and Kanfer, 2009), subjective self-report scales (e.g., Visual Analogue Scale for Fatigue, NASA-TLX) (Jacquet et al., 2021; Kunasegaran et al., 2023; Li et al., 2024), and physiological or neural measures, most notably EEG markers such as increased theta and alpha power, or altered event-related potentials (Tran et al., 2020; Trejo et al., 2015; Kunasegaran et al., 2023).

In the context of neurorehabilitation, mental fatigue is particularly important because it can impair task performance and learning (Li et al., 2024). Stroke patients are especially susceptible to fatigue (Paciaroni and Acciarresi, 2019) while also experiencing motor impairments (Raghavan, 2015), which may affect rehabilitation outcomes and motivate the use of brain computer interface (BCI)-based approaches.

BCIs translate neural activity into control signals that can support motor training when movement is limited and are increasingly used in motor neurorehabilitation (Tonin et al., 2025). In this context, motor imagery (MI) is a commonly used approach in which individuals mentally rehearse movements to engage sensorimotor networks in BCI-based neurorehabilitation (Tonin et al., 2025).

Numerous studies have shown that MI can enhance motor function in post-stroke patients (Nakintu et al., 2025; Tonin et al., 2025). It is a cognitive process in which an individual consciously simulates a specific movement internally, activating neural networks involved in motor planning and execution while intentionally inhibiting any actual motor output (Mulder, 2007). Prolonged sequences of MI have been shown to induce mental fatigue, leading to reduced alertness, slower information processing, and increased subjective effort (Li et al., 2024; Jacquet et al., 2021; Talukdar et al., 2019). Using magnetoencephalography (Shigihara et al., 2013) showed that fatigue accumulation differs between tasks with high and low cognitive demands and is associated with distinct changes in neural activity, indicating that fatigue-related processes depend on task-specific requirements. This was also confirmed in analysis of the EEG spectra data (Tanaka et al., 2012). These findings suggest that mental fatigue is inherently task-dependent and cannot be explained solely by time-on-task, although prolonged task duration remains an important contributing factor (Wascher et al., 2014). This highlights the importance of optimizing training environments to better support engagement and manage cognitive load during MI-based BCI tasks.

To mitigate challenges associated with MI-based BCI training, head-mounted display (HMD)–based virtual environments have been introduced to make training more interactive and adaptable (Fregna et al., 2023; Rosipal et al., 2022). The use of immersive HMD-based virtual reality (VR) in neurorehabilitation is growing, driven by its ability to enhance engagement and provide controlled conditions that approximate real-world training scenarios (Fregna et al., 2023).

Although immersive virtual reality delivered through an HMD can enhance engagement and improve BCI performance, prolonged interaction with HMD VR systems also imposes substantial cognitive and perceptual demands on users (Juliano et al., 2022). Sustained HMD use requires continuous sensorimotor integration, spatial orientation, and visual attention, which increases cognitive load and may induce discomfort over time (Juliano et al., 2022). These VR-related demands are associated with adverse effects such as cybersickness, manifested by nausea, dizziness, or vestibular instability, as well as visual strain (Biswas et al., 2024). The combination of sustained attention requirements and increased cognitive load may contribute to the development of mental fatigue and related adverse effects (Kunasegaran et al., 2023).

When HMD VR and MI paradigms are combined, as is common in immersive neurorehabilitation protocols (Fregna et al., 2023; Aderinto et al., 2023), these sources of mental fatigue can be confounded. The cognitive load imposed by the immersive environment (Breves and Stein, 2023) may interact with the sustained effort required for MI (Talukdar et al., 2019), potentially amplifying overall fatigue effects (Goodman et al., 2025) and making it difficult to disentangle their individual contributions. Such compounded mental fatigue can negatively impact user experience, reduce the reliability of neural signals (Talukdar et al., 2019), and limit the effectiveness of adaptive training strategies (Juliano et al., 2022). Therefore, understanding how mental fatigue develops over time from both VR usage and MI performance, and how it is reflected in behavioral and EEG measures, is essential for designing adaptive BCI–HMD systems that can sustain performance and support long-term rehabilitation outcomes.

A particular challenge arises when estimating mental fatigue during MI-based tasks. In MI paradigms, the primary neural biomarkers are typically associated with alpha and beta band changes over the sensorimotor cortex (Pfurtscheller, 2000). While previous research on mental fatigue has identified several EEG biomarkers, including alterations in oscillatory activity across multiple bands, with increased power in theta and lower alpha being most diagnostic (Tran et al., 2020; Trejo et al., 2015). Brain regions most commonly affected include the visual cortices and the frontal cortex (Kunasegaran et al., 2023). Consequently, fatigue-related neural dynamics may overlap with task-related sensorimotor activity, potentially confounding both processes. This overlap can not only impair accurate fatigue estimation but may also degrade the reliability of MI detection itself, thereby affecting closed-loop BCI performance and rehabilitation outcomes. Therefore, it is essential to disentangle MI-related sensorimotor activation from fatigue-related neural changes. In this work, we propose an approach that explicitly removes sensorimotor contributions from the model via component deflation, enabling a clearer separation of motor imagery processes from fatigue-related activity in the EEG signal.

The long-term aim of this research is to enable online detection of mental fatigue during MI-based neurorehabilitation, allowing future BCI-HMD systems to adapt task difficulty, pacing, or feedback to maintain engagement and optimize therapeutic outcomes. As a step toward this goal, the present study investigates EEG-based monitoring of mental fatigue during prolonged MI sessions within BCI-HMD systems and evaluates the feasibility of real-time classification under artifact-prone conditions resembling clinical environments. This research is particularly relevant for post-stroke motor neurorehabilitation, where consistent and sustained motor MI training is essential for functional recovery.

## 2 Methods

### 2.1 Experimental design

The experimental study was designed to investigate the feasibility of EEG-based monitoring of mental fatigue during prolonged MI training in immersive BCI-HMD virtual-reality environments.

The protocol followed a structured three-session design, enabling within-subject comparisons between MI and control conditions. We hypothesized that sustained BCI-HMD use during the MI paradigm would lead to measurable neural indicators of mental fatigue, reflected in progressive changes in occipital and sensorimotor alpha activity.

The BCI-HMD virtual-reality environment used in this study is described in detail in Rosipal et al. (2022). The experiment consisted of three distinct sessions conducted on three separate days:

1. *Mirror-box session*- The first session was conducted to obtain individualized “atoms,” defined as elementary oscillatory rhythms characterized by spatial and frequency weight vectors. These atoms were extracted using tensor decomposition of EEG data recorded while participants performed instructed upper-limb movements within a mirror-box paradigm. The resulting atoms were subsequently used as frequency–spatial filters for MI detection during the VR session (Rosipal et al., 2019). The mirror-box paradigm (Mulder, 2007) has been shown to engage motor imagery and action-observation networks and to support motor rehabilitation.
2. *MI VR-HMD session*- Participants engaged in a MI paradigm in which they mentally imagined performing gestures, without any overt physical movement, in response to auditory voice commands.
3. *Control VR-HMD session*- This condition was used to isolate the visual and attentional effects of performing a VR-based task from the cognitive load associated with MI. Participants were presented with the same VR stimuli as in the MI session but were instructed to observe the task passively without engaging in MI and to count the number of successful trials. The Control BCI-HMD condition was designed as a sham experiment, in which successful trials were presented randomly and were not controlled by EEG activity. To maintain comparable engagement, a proportion of trials was randomly designated as successful with probabilities of 0.30, 0.40, and 0.35 for the three blocks.

#### 2.1.1 Pre-session requirements

To minimize baseline fatigue and maintain physiological consistency across sessions, participants were instructed to adhere to a standardized set of pre-session conditions:

- Sleep at least 8 h the night before the session.
- Avoid cognitively demanding tasks on the day of testing. Examples of such tasks were given to explain cognitive demand, including taking exams, prolonged computer work or extensive gaming activity.
- Refrain from alcohol and drug consumption for at least 24 h prior to testing.
- Consume a meal 1.5–2 h before the session.
- Avoid nicotine consumption for at least 3 h prior to testing.

Compliance with these requirements was confirmed using a pre-test checklist upon arrival.

#### 2.1.2 Session structure

**Figure 1:**
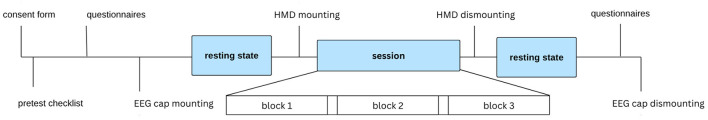
Diagram of the full experimental procedure.

**Figure 2:**
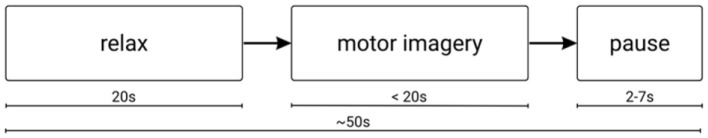
Schematic of a single trial in the VR session, showing the resting-state (relax) period and motor imagery phases, followed by a pause.

Both the MI and control sessions followed a standardized structure to ensure procedural consistency (see Figure 1). Upon arrival, participants reviewed and signed the informed consent form, followed by completion of a pre-test checklist to confirm compliance with the pre-session requirements. They then completed pre-session questionnaires. Next, an eyes-closed (EC) and eyes-open (EO) resting-state EEG recording was obtained. Participants were then fitted with the HMD, and the VR experimental phase—either the MI or control session–was conducted. After the VR task, the HMD was removed, and a post-session EC and EO resting-state recording was performed following the same protocol as in the pre-session phase. Finally, participants completed post-session questionnaires assessing fatigue and discomfort, and the EEG cap was removed. Both sessions consisted of three blocks of 25 trials each. Each trial, illustrated in Figure 2, began with a 20-second resting-state period, which was used to set a threshold for the subsequent MI phase. In the MI session, the resting-state was followed by a MI task. When the required neural patterns were detected, a virtual arm animation was triggered, reaching toward an object. If no activation was detected, the trial automatically terminated after 20 seconds. After the MI phase, a pause with a randomized duration between 2 and 7 seconds was included. In the control session, the animation was activated randomly, independent of neural activity. Each trial lasted approximately 50 seconds, and each session lasted around one hour.

#### 2.1.3 Motor imagery task

The MI task involves the mental simulation of movements without actual physical execution. MI engages overlapping brain regions that are active during actual movement, including the primary motor cortex and supplementary motor areas. Because the sensory and motor cortices exhibit dynamic neuroplasticity, MI is a critical component of motor learning and recovery, particularly in post-stroke rehabilitation. Consequently, both mirror-box therapy and MI can play a key role in improving motor function in stroke patients (Mulder, 2007; Rosipal et al., 2019).

During the task, participants were instructed to imagine executing a predefined gesture—reaching with their hand to pick up a cup—whenever prompted by an audio cue within the HMD, while focusing on the kinesthetic sensations associated with the movement.

### 2.2 Participants

EEG data were collected from 15 participants during the initial mirror-box experiment. All participants provided informed consent for each session in which they participated and received monetary compensation. Exclusion criteria included color vision deficiencies, current medication use, diagnosed psychiatric disorders, and motor impairments. Application of these exclusion criteria was based on self-report without formal clinical screening.

Due to participant dropouts, additional data were collected from only nine participants in the MI session and ten in the control session, with eight completing all three sessions. One participant was excluded from the final analysis due to excessive EEG artifacts and a self-reported psychiatric disorder, resulting in a total of seven participants included in the analysis.

The remaining seven participants comprised three males and four females, with an average age of 22.86 ± 3.44 years (range: 19–30). According to self-reports, all participants were right-handed, three reported prior experience with VR, and none reported prior exposure to BCI. All participants had normal or corrected-to-normal vision and reported no history or current experience of motion sickness or motor disabilities.

### 2.3 Self-reporting methods

Subjective fatigue was assessed using a custom-made questionnaire comprising seven items targeting symptoms commonly associated with visual discomfort, motion-induced instability, and general fatigue during immersive virtual reality tasks.

Participants rated each item on a 7-point scale, where 1 indicated “Not at all” and 7 indicated “Yes, very much.” The full list of questionnaire items is presented in Table 1. The assessment was administered at multiple time points: before the experiment, between blocks of the VR sessions, and immediately after task completion, allowing for the examination of changes in subjective fatigue throughout the experimental procedure.

**Table 1:** Custom fatigue questionnaire used to assess subjective fatigue.

Item	Question
1	Are you feeling nauseous (for example, feeling sick)?
2	Do you experience dizziness (for example, lightheadedness or a sensation of spinning, or a feeling of floating in space)?
3	Do you feel insecure, a perceived loss of stability?
4	Do you experience eye fatigue or a feeling of drowsiness?
5	Do you experience eye strain, blurred vision, or headache?
6	Are you feeling tired?
7	Are you feeling motivated for your next session/Do you feel motivated to continue training?

Ratings were provided on a 7-point scale (1 = “Not at all,” 7 = “Yes, very much”).

Spearman's rank correlation was used to evaluate the relationship between individual questionnaire items and the fatigue estimates (see Section 2.7.1), as it captures monotonic associations without assuming linearity and is appropriate for ordinal questionnaire responses.

### 2.4 EEG activity

EEG activity was continuously recorded using the BCI-HMD system. Signals were acquired with the wireless g.Nautilus PRO FLEXIBLE system, an FDA-cleared device equipped with 32 Ag/AgCl wet electrodes positioned according to the international 10–20 system. The EEG cap was mounted in accordance with the manufacturer's specifications.

### 2.5 Virtual reality and head-mounted display

The virtual reality environment was presented using a Meta Quest 2 (Meta Platforms, Inc., 2020) standalone VR headset. The device features a resolution of 1,832 × 1,920 pixels per eye, a refresh rate of up to 120 Hz, and integrated inside-out tracking for six degrees of freedom (6DoF) in head and hand movements. The VR application was developed using the *A-Frame* software platform and deployed on the headset, the implementation was previously reported in Rosipal et al. (2022).

### 2.6 Data processing

The EEG recordings were first converted to EDF format and preprocessed in Brain Vision Analyzer 2.3.03 (Brain Products GmbH, 2023). Preprocessing consisted of the following steps: (a) identification and interpolation of channels with missing or problematic data; (b) application of Independent Component Analysis (ICA) to detect blink-related artifacts; (c) a semi-automatic threshold-based procedure to identify general artifacts, followed by visual inspection to ensure accurate marking; and (d) an additional step to detect EMG artifacts prior to data export.

The data were subsequently processed in MATLAB (The MathWorks, Inc., 2024), where the signals were re-referenced to the common average and a 1.5 Hz high-pass filter was applied. Continuous EEG data were segmented into 2 s epochs with a 500 ms moving window, followed by application of a 50 Hz notch filter. Features—including fractal, harmonic, and raw spectral components—were then extracted from these epochs using an Irregular-Resampling Auto-Spectral Analysis (IRASA) -based approach (Wen and Liu, 2016) with a Hann window. In this study, we focused on the harmonic component of the spectrum.

### 2.7 Model settings for classification

After preprocessing, an individual model was trained for each participant. The goal of the classification procedure was to discriminate between two different levels of mental fatigue based on EEG features. To leverage the multidimensional structure of the EEG data, the classification was performed using the N-way Partial Least Squares (N-PLS) algorithm. This method was chosen for its ability to model structured, high-dimensional neural data and to extract latent variables relevant for classification.

#### 2.7.1 N-way Partial Least Squares

The N-PLS algorithm is a supervised multivariate regression technique that generalizes conventional Partial Least Squares to multi-way data structures (Bro, 1996). It achieves this by projecting the input tensor **X** and the response tensor **Y** onto a shared low-dimensional latent subspace that maximizes the covariance between their respective latent representations.

Resulting models are defined by their latent representations—often referred to as components, atoms, or factors—which are described by temporal, spectral, and spatial weights. These weights indicate the importance of specific locations, frequencies, and time points, and how they contribute to the model's predictions.

Various Partial Least Squares (PLS) methods have been applied to EEG data, demonstrating their ability to capture spatial, spectral, and temporal patterns simultaneously (Eliseyev and Aksenova, 2013; Trejo et al., 2015). This makes N-PLS particularly well-suited for BCI and fatigue research, where neural signals exhibit high dimensionality across electrodes, frequency bands, and time points.

In this study, the tensor **X** represents the multidimensional EEG data organized across time, frequency, and spatial modes, while the matrix **Y** encodes class labels, where 0 corresponds to low fatigue and 1 to high fatigue. The N-PLS model extracts latent variables that capture the shared variance between the EEG features and the corresponding fatigue states, providing a framework for identifying neural markers of mental fatigue in complex BCI-HMD VR experiments.

The relationship between **X** and **Y** can be expressed by Equations 1–4.


$$\begin{array}{l} \text{X}=\overset{F}{\underset{f=1}{\sum }}{\text{t}}_{f}○{\text{w}}_{f1}○{\text{w}}_{f2}○\cdots ○{\text{w}}_{fN}+\text{E}, \end{array}$$ (1)

$$\begin{array}{l} \text{Y}=\overset{F}{\underset{f=1}{\sum }}{\text{u}}_{f}{\text{q}}_{f}^{\text{T}}+\text{C}, \end{array}$$ (2)

where ° denotes the outer product (Cichocki et al., 2009), **t**_*f*_ and **u**_*f*_ are the latent score vectors for **X** and **Y**, **w**_*fn*_, and **q**_*f*_ are mode-specific weight vectors, and **E** and **C** represent residuals. The latent variables are estimated to maximize the covariance


$$\begin{array}{l} \underset{{\text{t}}_{f},{\text{u}}_{f}}{\max}\;\text{cov}\left({\text{t}}_{f},{\text{u}}_{f}\right) \end{array}$$ (3)

between **t**_*f*_ and **u**_*f*_.

The overall regression model linking the latent representation of **X** to **Y** is defined as:


$$\begin{array}{l} \text{Y}=\text{T}\text{B}+\text{H}, \end{array}$$ (4)

where **T** = [**t**_1_, …, **t**_*F*_] contains the latent score vectors, **B** denotes the matrix of regression coefficients, and **H** represents the residual matrix.

The number of factors *F* determines the dimensionality of the latent subspace. In this study, combinations of atoms that yielded the highest test-set accuracies were selected for the final model, with a maximum of *F* = 4. The extracted latent components capture the shared variance between the EEG tensor and fatigue class labels, enabling the identification of neural patterns associated with different levels of mental fatigue.

Each model was trained using artifacts free resting-state EEG spectral features, recorded both pre- and post-session, within the 5–20 Hz range and processed according to the pipeline described in Section 2.6.

#### 2.7.2 Cross-validation and general model creation

Initially, a five-fold cross-validation procedure was performed to optimize model parameters and evaluate generalization performance. No additional class balancing or sampling strategies were applied during model training. In each iteration, four folds were used for training and one for testing. The model was fitted on the combined dataset from both the MI and control sessions to obtain a more generalized representation of individualized neural patterns. After completing all five iterations, the resulting N-PLS models were compared across folds. Because highly similar latent patterns consistently emerged, the corresponding components were averaged to create a general atom, representing a stable and generalized participant-specific aspect of fatigue-related neural activity. This aggregation step reduced the influence of fold-specific variability and provided a more robust representation of how fatigue manifests in the individual's EEG data. The long-term objective of this approach is to enable deployment of such models on real-time EEG streams for continuous fatigue monitoring during rehabilitation therapy.

Upon examining the frequency-domain weights and corresponding electrode topographies, it was observed that, in certain cases, the model captured traces of task-related sensorimotor activity. The μ rhythm, widely studied in EEG MI research, typically occurs in the 8–12 Hz frequency band over the sensorimotor region and decreases during both motor execution and motor imagery. Training of the μ rhythm is therefore beneficial for neuromotor rehabilitation (Mulder, 2007; Chen et al., 2021).

In our initial models, overlapping patterns associated with the MI task were identified, corresponding to typical signatures of sensorimotor rhythm modulation. These observed patterns matched the participant's individual μ atom extracted from EEG data recorded during the mirror-box session using the PARAFAC (Parallel Factor Analysis) method (Harshman, 1970; Cichocki et al., 2009). PARAFAC is an unsupervised multi-way decomposition technique that generalizes matrix factorization to higher-order arrays (tensors). The presence of μ-like patterns likely reflects a neuroplasticity effect resulting from repeated MI. Because these components reflected motor execution or imagery processes rather than fatigue-related activity, their presence in the model was undesirable.

**Figure 3:**
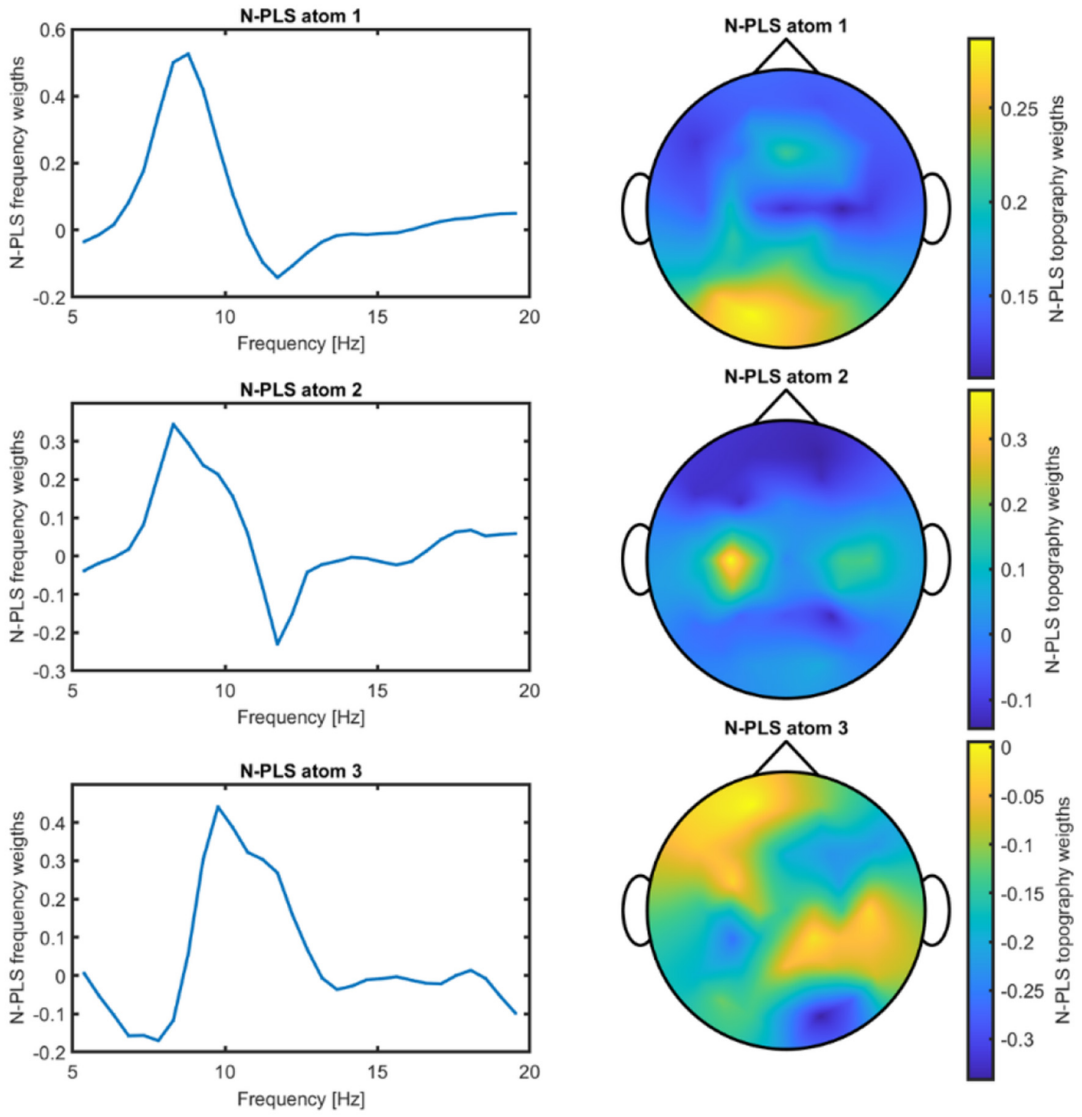
Frequency and spatial weight distributions of the N-PLS model components for Participant 05.

An example is shown in Figure 3, where N-PLS atom 2 captured elements of task-related sensorimotor activation rather than purely fatigue-related variance. This atom exhibited maximal spatial weights over the left sensorimotor cortex together with dominant spectral contributions in the alpha band, a hallmark of μ-rhythm modulation during motor imagery.

**Figure 4:**
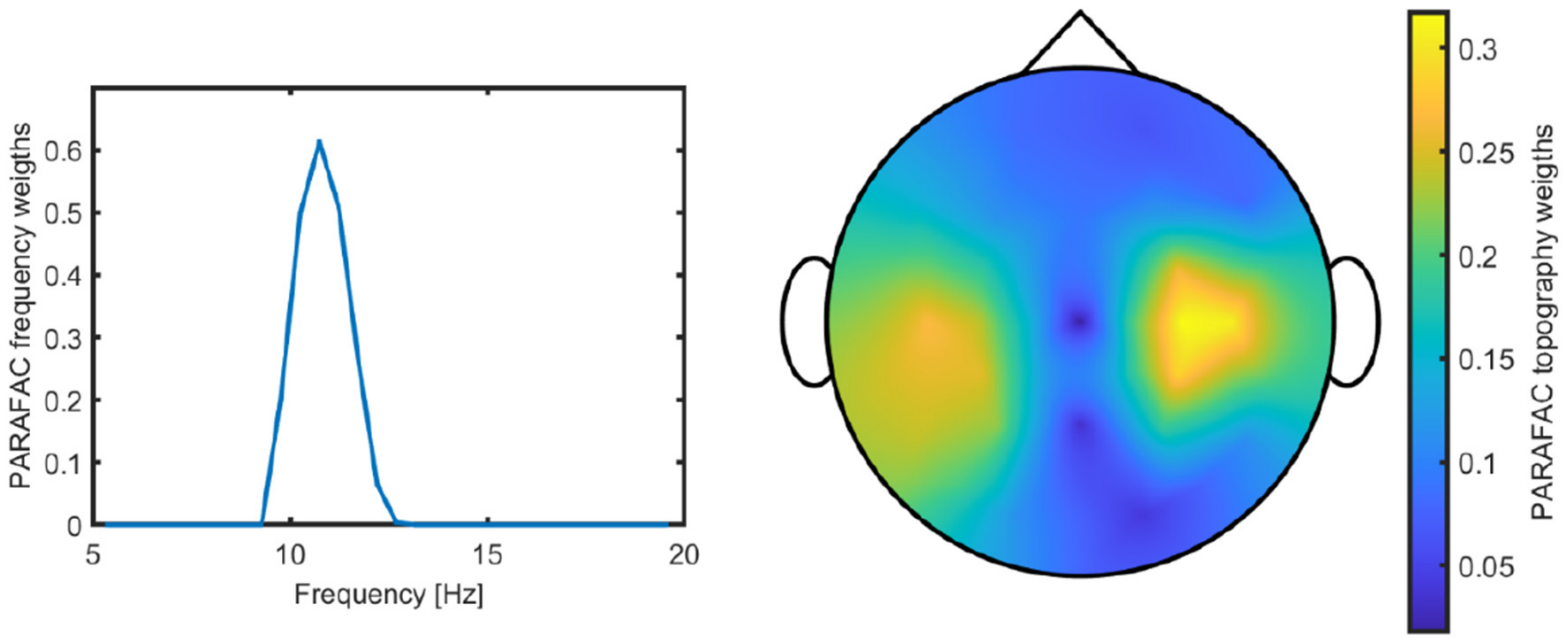
PARAFAC-derived μ component for Participant 05, showing its characteristic spatial distribution over the sensorimotor cortex and dominant frequency around 10.75 Hz.

This effect was particularly evident when comparing the “μ-like” components extracted by N-PLS with the μ components that participants were trained to modulate in the VR environment. Figure 4 illustrates the PARAFAC-derived μ atom for Participant 05, with the component's peak frequency occurring at 10.75 Hz.

#### 2.7.3 μ atom deflation

Because both the “μ-like” N-PLS component and the trained μ rhythm exhibit activity in the sensorimotor cortex with prominent spectral power around 10.75 Hz, and to ensure that the N-PLS model captured neural signatures of fatigue rather than task-related or training-induced activity, the PARAFAC-derived μ components were excluded from the final model.

Since both the μ component and the N-PLS model operate on tensor-structured EEG data, the identified μ atom was deflated from the EEG data (Cichocki et al., 2009; Roštáková and Rosipal, 2025). Specifically, the trained μ rhythm was removed, effectively eliminating its contribution to the model, as expressed by Equation 5:


$$\begin{array}{l} {\text{X}}_{\text{deflated}}=\text{X}-{\text{t}}_{r}○{\text{w}}_{r}^{\left(2\right)}○{\text{w}}_{r}^{\left(3\right)} \end{array}$$ (5)

where **t**_*r*_ is the mode-1 score vector representing the temporal profile of the component, while ${\text{w}}_{r}^{\left(2\right)}$ and ${\text{w}}_{r}^{\left(3\right)}$ are the mode loadings representing the spatial and frequency profiles, respectively.

The resulting residual tensor, **X**_deflated_, was then used for prediction. Because the μ atom was extracted using 10 central electrodes to ensure stability and robustness for online application, both frequency and spatial weights were zero-padded to enable proper deflation.

**Figure 5:**
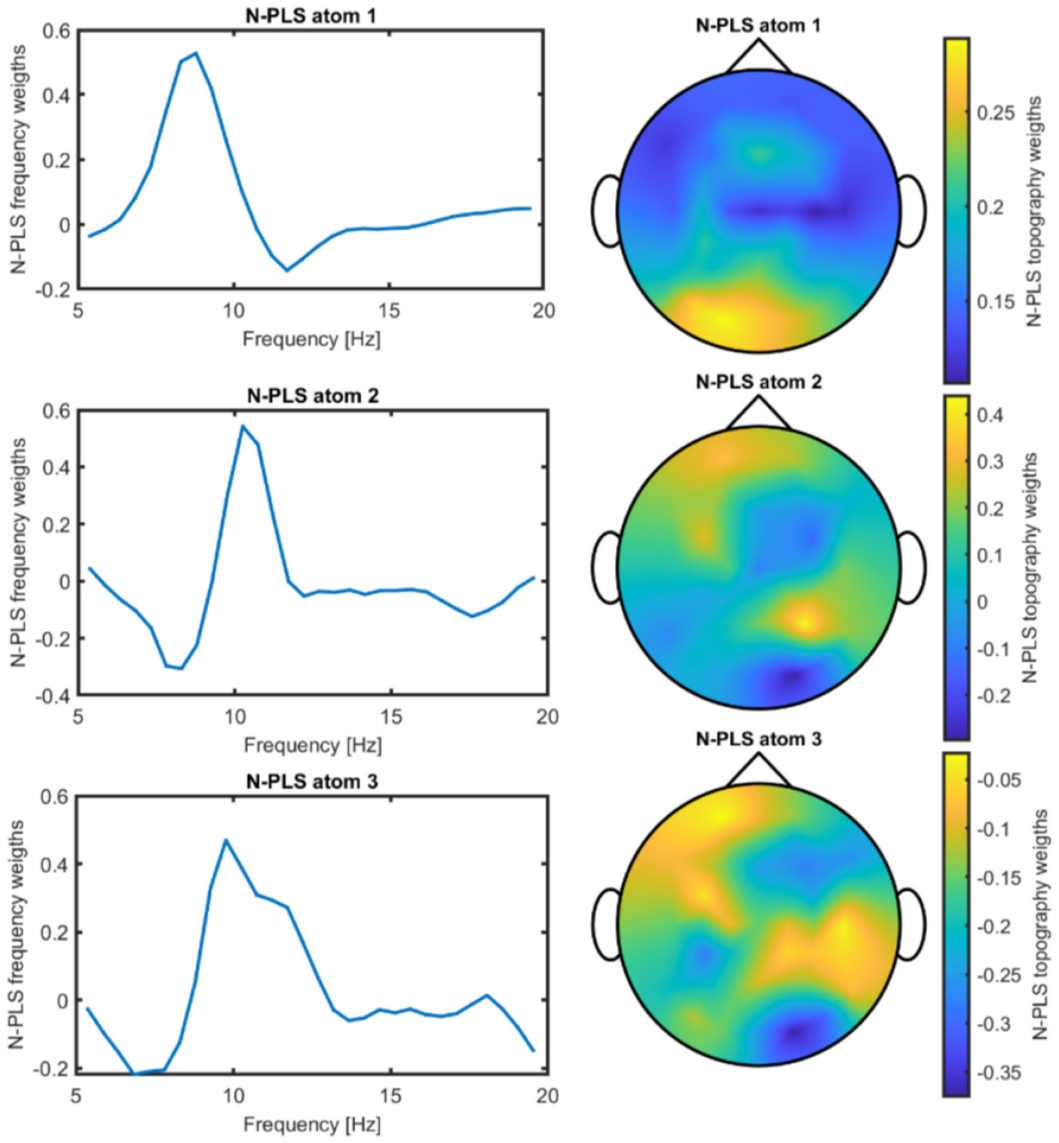
Frequency and spatial weight distributions of the N-PLS model components after deflation of the PARAFAC μ atom for Participant 05.

After deflating the μ component, the N-PLS model was retrained. An example of a resulting model is shown in Figure 5. Comparison of the two models shown in Figures 3, 5—N-PLS with and without the μ activity contribution—demonstrates that the model remains robust and that the observed changes can be attributed to the deflation of task-specific activity.

#### 2.7.4 Classification and evaluation

Following construction of the model with deflated μ activity, linear discriminant analysis (LDA) was employed to classify the data and generate probabilistic outputs. Classification was performed on harmonic spectral data preprocessed as previously described, but without artifact removal. Fatigue predictions were estimated on the artifactual data, with the goal of developing a robust system suitable for integration into an online fatigue monitoring framework.

For performance assessment, classification accuracy was evaluated across cross-validation folds as a measure of overall correctness. Precision, recall, and F1 score were also computed to provide a more detailed evaluation of classification performance. Precision reflects the proportion of correctly identified positive samples among all predicted positives, while recall quantifies the proportion of true positive samples that were correctly detected. The F1 score represents the harmonic mean of precision and recall, balancing false positive and false negative errors. Fatigue estimates were analyzed on the test set for each participant to assess model generalization at the individual level.

In addition, the Polygon Area Metric (PAM) was computed to provide a single comprehensive measure of classifier performance by jointly considering multiple evaluation metrics, including classification accuracy (CA), sensitivity (SE), specificity (SP), area under curve (AUC), Jaccard index (JI), kappa (K), and F-measure (FM) (Aydemir, 2021).

## 3 Results

### 3.1 Subjective measures analysis

Descriptive statistics for the subjective questionnaire measures are summarized in Table 2. Overall levels of nausea, dizziness, and vestibular instability remained unchanged throughout the experiment, with mean values close to the lower bound of the scale. Visual fatigue, visual discomfort, and mental fatigue showed a moderate increase, indicating a noticeable yet manageable level of strain during the task. Participant motivation remained high, with a mean score above 5, suggesting that engagement and willingness to continue training were maintained despite the presence of some fatigue-related symptoms.

**Table 2:** Descriptive statistics for the subjective questionnaire measures.

Variable	Mean	SD	Median
Vestibular instability	1.33	0.69	1
Nausea	1.30	0.68	1
Dizziness	1.55	0.99	1
Motivation	5.38	1.62	5
Visual fatigue	2.84	1.25	2
Visual discomfort	2.27	1.44	2
Mental fatigue	2.81	1.21	3

**Figure 6:**
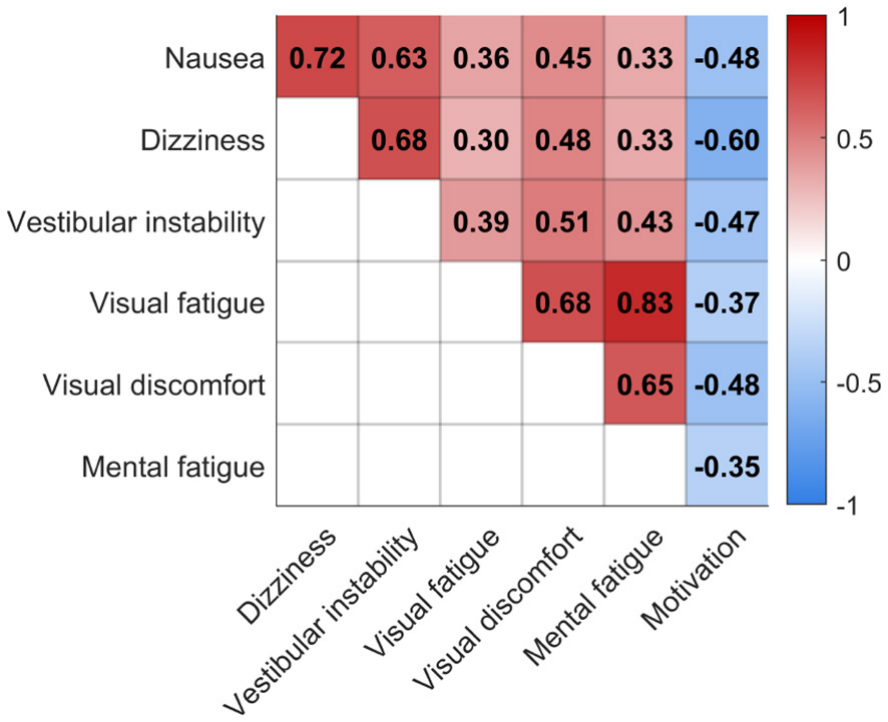
Correlation matrix of subjective fatigue measures, illustrating the strength and direction of associations among all questionnaire items.

Correlation analysis was conducted using Spearman's rank correlation. The analysis revealed clear associations among subjective fatigue measures. Visual and mental fatigue were closely related, as were nausea and dizziness. Moderate positive relationships were also observed among visual discomfort, vestibular instability, and other fatigue dimensions. In contrast, dizziness showed a negative association with motivation, suggesting that increased vestibular symptoms were linked to reduced willingness to continue the task, as shown in Figure 6.

### 3.2 EEG analysis

After analyzing the subjective measures obtained from questionnaires administered during the experiment, we proceeded to examine objective indicators derived from EEG data.

Our analysis focused on the harmonic component of the EEG spectrum, an established framework for assessing brain states and fatigue-related changes, as oscillatory activity in specific frequency bands has been shown to reflect cognitive performance and mental fatigue (Klimesch, 1999, 2012; Tran et al., 2020). Within this representation, we concentrated on the alpha frequency range, as it is a dominant spectral peak and fatigue index in resting-state EEG. Although previous studies have also reported fatigue-related effects in the theta and beta bands, our models were trained and evaluated on resting-state segments, where alpha-band dynamics are expected to exhibit the strongest modulation.

**Figure 7:**
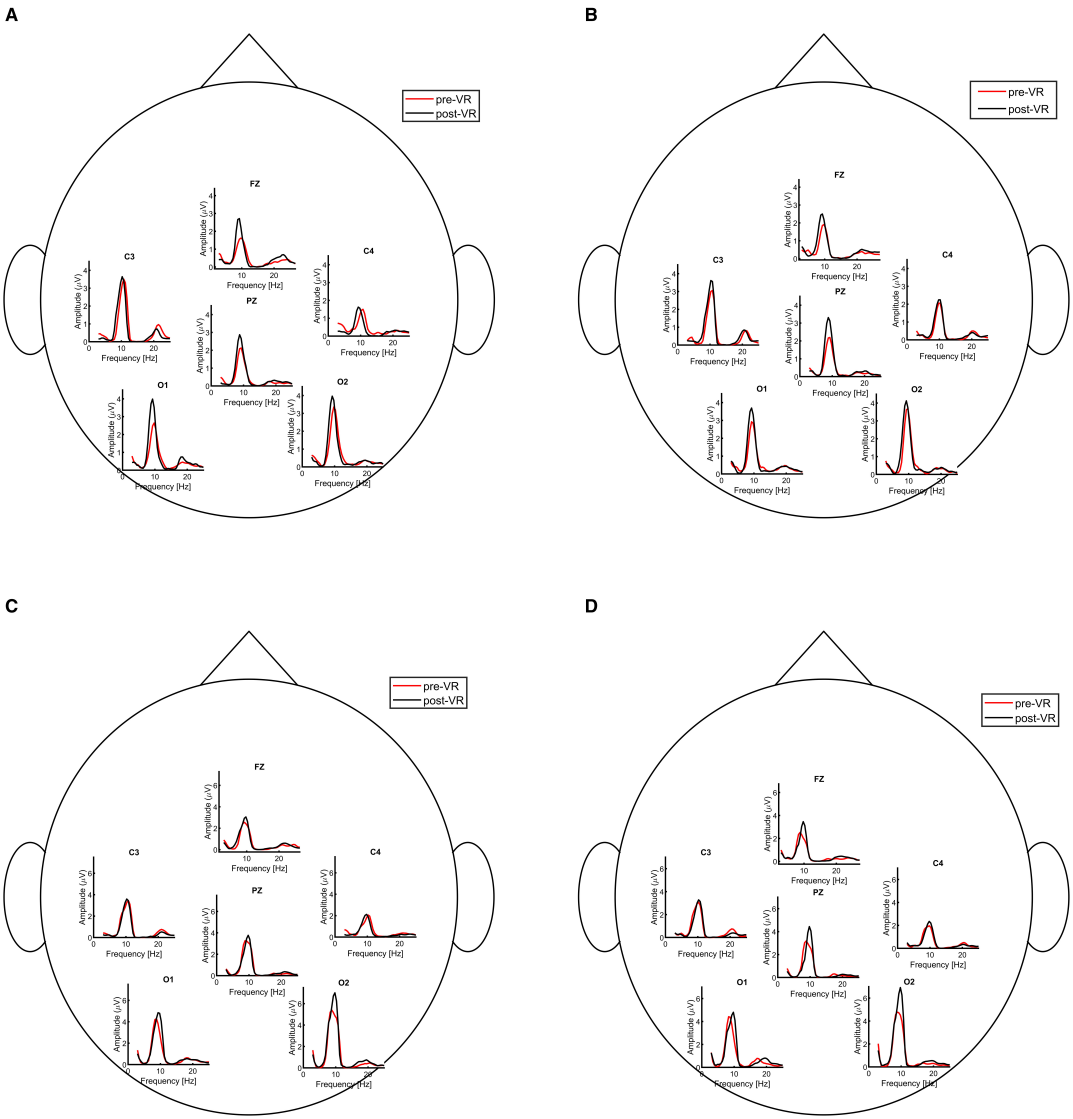
Harmonic spectra of pre- and post-session resting-state EEG for Participant 05. The panels show, respectively: eyes-open MI session, eyes-open control session, eyes-closed MI session, and eyes-closed control session. Data are shown for electrodes O1, O2, C3, C4, Fz, and Pz; the analysis was conducted across all recorded channels. **(A)** Eyes open MI session. **(B)** Eyes open control session. **(C)** Eyes closed MI session. **(D)** Eyes closed control session.

First, we analyzed the harmonic spectra of pre- and post-session EEG recordings under both EC and EO conditions, as illustrated in Figure 7. As expected, overall spectral amplitudes were higher in the EC condition than in the EO condition, a phenomenon known as the Berger effect (Berger, 1929). Furthermore, post-session recordings exhibited greater amplitudes than pre-session recordings. While Figure 7 presents data from channels O1, O2, C3, C4, Fz, and Pz, the analysis was conducted across all recorded channels.

**Figure 8:**
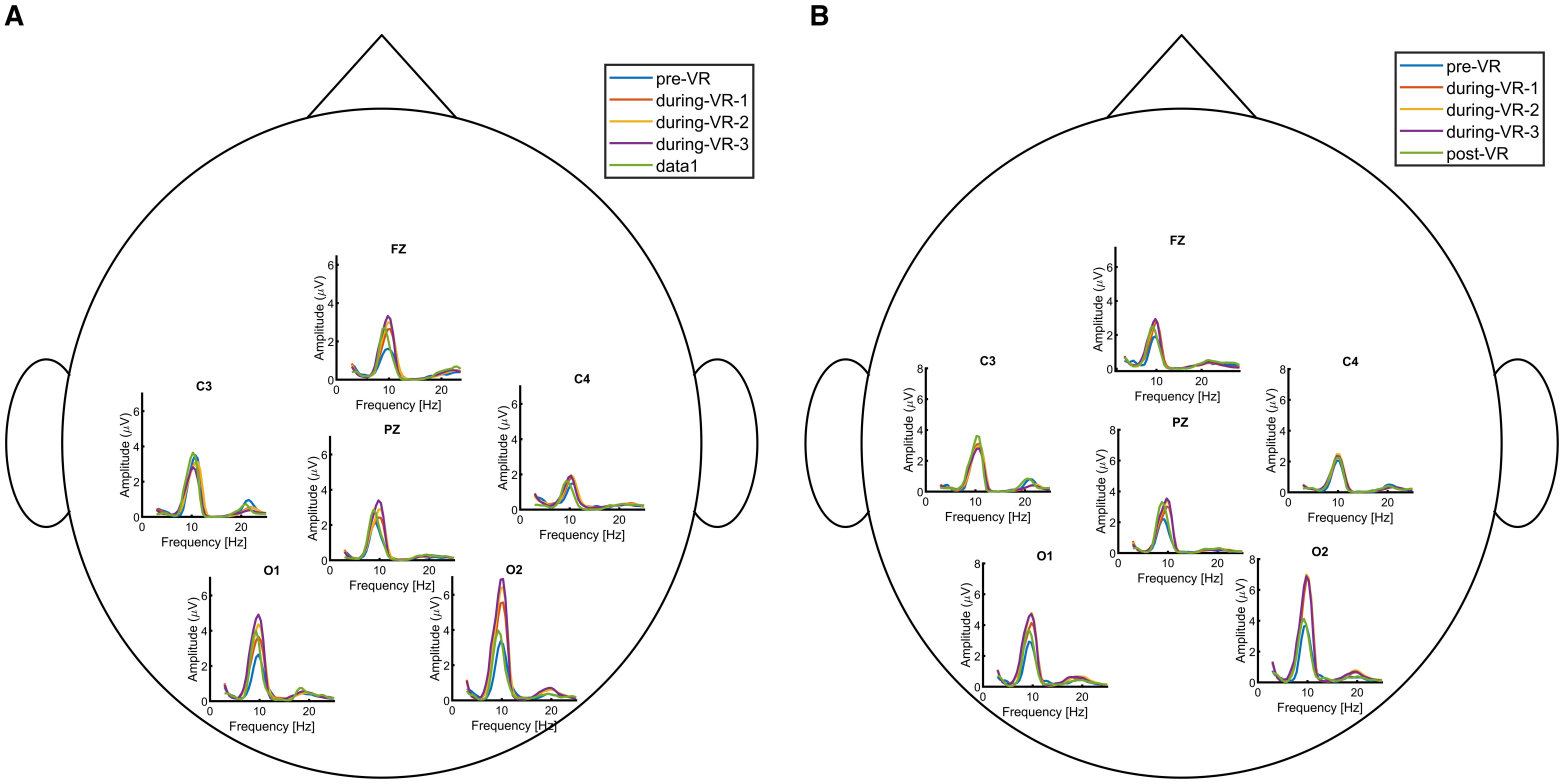
Harmonic spectra for Participant 05 across five conditions: pre-session EO, VR blocks 1–3, and post-session EO. Data are shown for electrodes O1, O2, C3, C4, Fz, and Pz, while the analysis was conducted across all recorded channels. **(A)** MI session. **(B)** Control session.

Following the analysis of pre- and post-session resting-state data, we examined the harmonic spectra for each block of the resting-state within the VR session. Figure 8 shows five conditions: pre-session EO, three VR blocks, and post-session EO. During the MI session, a clear progression was observed across blocks, with spectral amplitude gradually increasing from block 1 to block 3, whereas this effect was less pronounced in the control session data.

EEG signals recorded during the VR session exhibited distinct spectral profiles compared to resting-state data. The potential effects of the VR environment and the HMD on these differences are discussed in Section 4.1.2.

**Figure 9:**
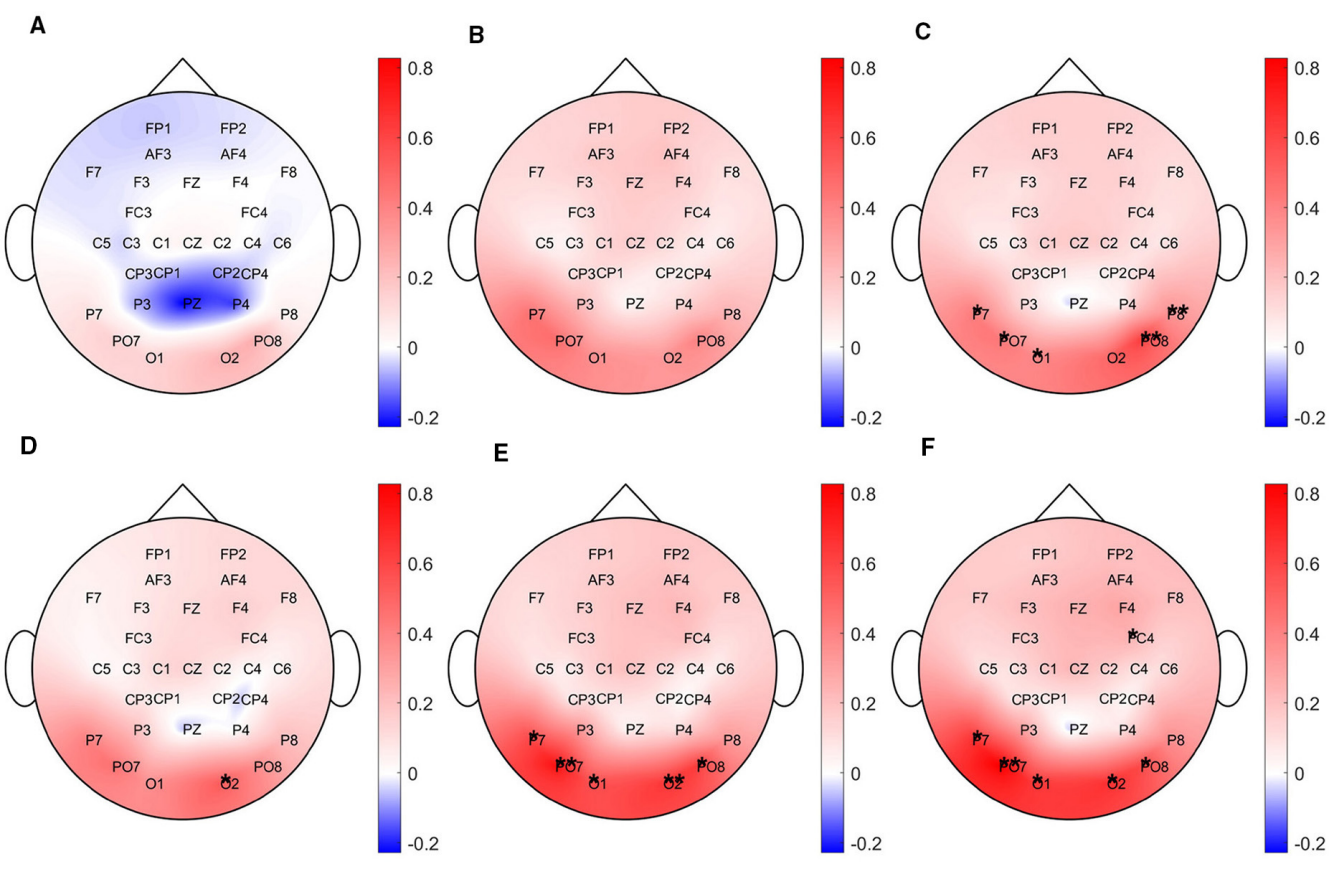
Grand-average spectra across different time points in the experiment for alpha band frequencies (8–12 Hz). The first row shows differences between the beginning session and VR blocks 1–3 for the MI session, while the second row shows the same for the control session. Asterisks (*) indicate electrodes showing statistically significant differences (*p* < 0.05). **(A)** MI session: VR block 1. **(B)** MI session: VR block 2. **(C)** MI session: VR block 3. **(D)** Control session: VR block 1. **(E)** Control session: VR block 2. **(F)** Control session: VR block 3.

For the final analysis of the EEG data, we examined the grand-average power spectra across different time points in the experiment, focusing on frequencies within 1 Hz of each participant's individually determined alpha frequency. As shown in Figure 9, electrodes associated with occipital and parietal alpha activity exhibited an increase in alpha power following the VR sessions.

**Figure 10:**
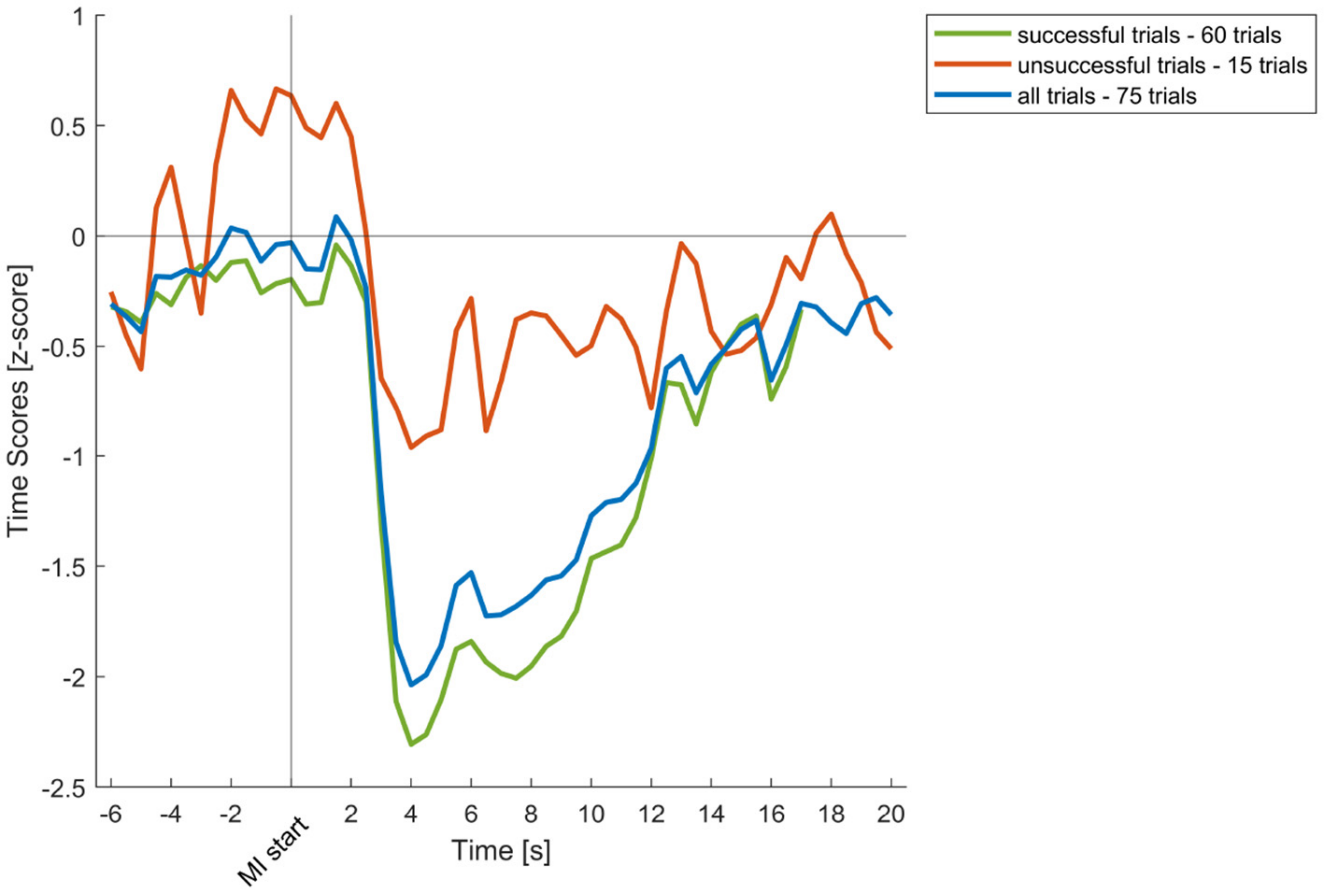
Desynchronization of μ-rhythm scores during the MI session for Participant 05. The figure shows the average of successful, unsuccessful, and all trials. Each trial was time-locked to the onset of the motor imagery command to examine event-related desynchronization patterns.

Additionally, we examined neural desynchronization, as reflected in the scores of the modulated μ atom during the MI session. In most cases, a clear desynchronization of μ activity was observed, with distinguishable differences between successful and unsuccessful trials. The average desynchronization across trials for Participant 05 is shown in Figure 10. Notably, even unsuccessful trials exhibited a noticable level of μ desynchronization.

### 3.3 Mental fatigue classification

First, we applied the N-PLS classification procedure to epochs extracted from EEG segments recorded during the pre- and post-VR session resting-state periods of seven participants. We associated data from the pre-session period with non-fatigued (low fatigue), while post-session data represent high levels of mental fatigue. Resting-state data were used exclusively to minimize the influence of task-related μ-rhythm and other associated cognitive modulations, which could otherwise bias the classification. EEG segments were divided into low-fatigue and high-fatigue classes, the dataset comprised 1,072 low-fatigue and 1,322 high-fatigue epochs with total number of epochs being 2,394.

**Figure 11:**
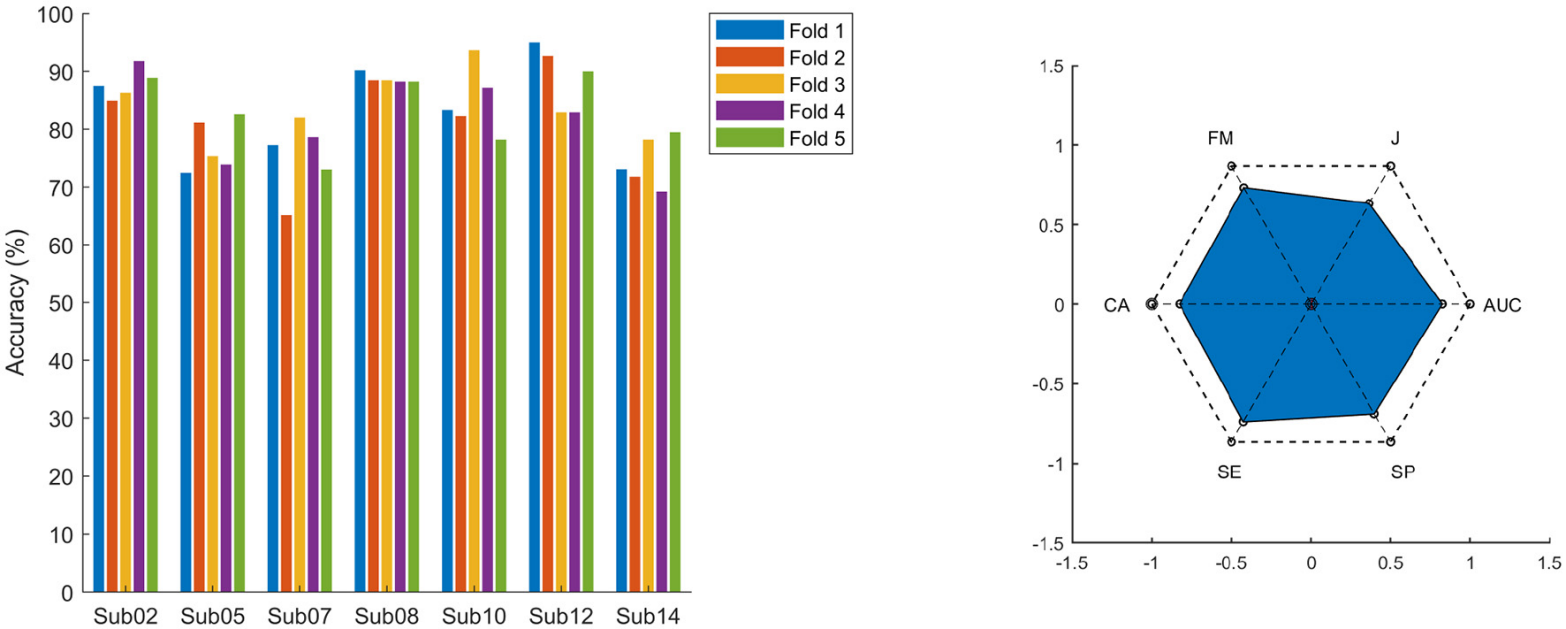
Classifier evaluation metrics. On the **left**: Classification accuracy for each participant across the five cross-validation folds. Each bar represents the accuracy for a single fold, illustrating fold-wise consistency and model robustness. On the **right**: Polygon Area Metric (PAM) for each participant across the entire dataset. PAM jointly considers multiple evaluation metrics: classification accuracy (CA), sensitivity (SE), specificity (SP), area under the curve (AUC), Jaccard index (JI), kappa (K), and F-measure (FM), summarizing them into a single score.

To assess model reliability, five-fold cross-validation was performed for each participant, and classification accuracy was calculated for each fold. Across folds, the within-participant standard deviation of accuracy ranged from 0.83% to 6.48%, indicating stable classification performance, as low standard deviations suggest consistent model behavior across folds. The bar plots in Figure 11 left illustrate classification accuracy for each participant across all folds. The figure on the right in Figure 11 shows the Polygon Area Metric (PAM) (Aydemir, 2021) graphs computed over the entire dataset, encompassing all participants, with an overall PAM value of 0.66.

Following deflation of the μ rhythm from the EEG data, the N-PLS model was retrained, and final classification accuracies were obtained. These accuracies reflect the model's ability to capture fatigue-related neural signatures independent of task-specific μ activity. The average classification accuracy was 83.49 ± 6.34%, where ±6.34% represents the standard deviation across participants.

For each participant, an N-PLS-LDA model was constructed, and the optimal model was selected as described above. The number of N-PLS components retained ranged from 3 to 4, with an average of 3.71 components. Specifically, two of the seven participants had three components in the final model, while the remaining participants had four. Each model consists of frequency and spatial electrode weights; an example can be seen in Figure 5. Electrode weights can be projected onto a 2D topographic map of the scalp, indicating which electrodes or brain regions contributed most strongly to classification.

To further interpret these individualized N-PLS-LDA models and the fatigue-related neural signatures they capture, we examined the separation of resting-state data within the learned N-PLS latent space and its evolution across recordings.

#### 3.3.1 Representation of data separation in the N-PLS latent space

Beyond classification performance, the N-PLS latent space provides an interpretable representation of the evolution of fatigue-related neural states across time and experimental conditions, even in the presence of artifacts.

Recall, that all data used in this analysis correspond to resting-state recordings. Resting-state periods provide a baseline neural activity measure that is less affected by task-related activity or cognitive engagement, making them particularly suitable for assessing mental fatigue.

For visualization purposes, the N-PLS components were projected into two- or three-dimensional latent spaces. Despite this restriction to the first two or three components, class separation remained generally evident, with data blocks occupying distinct regions within the latent space.

**Figure 12:**
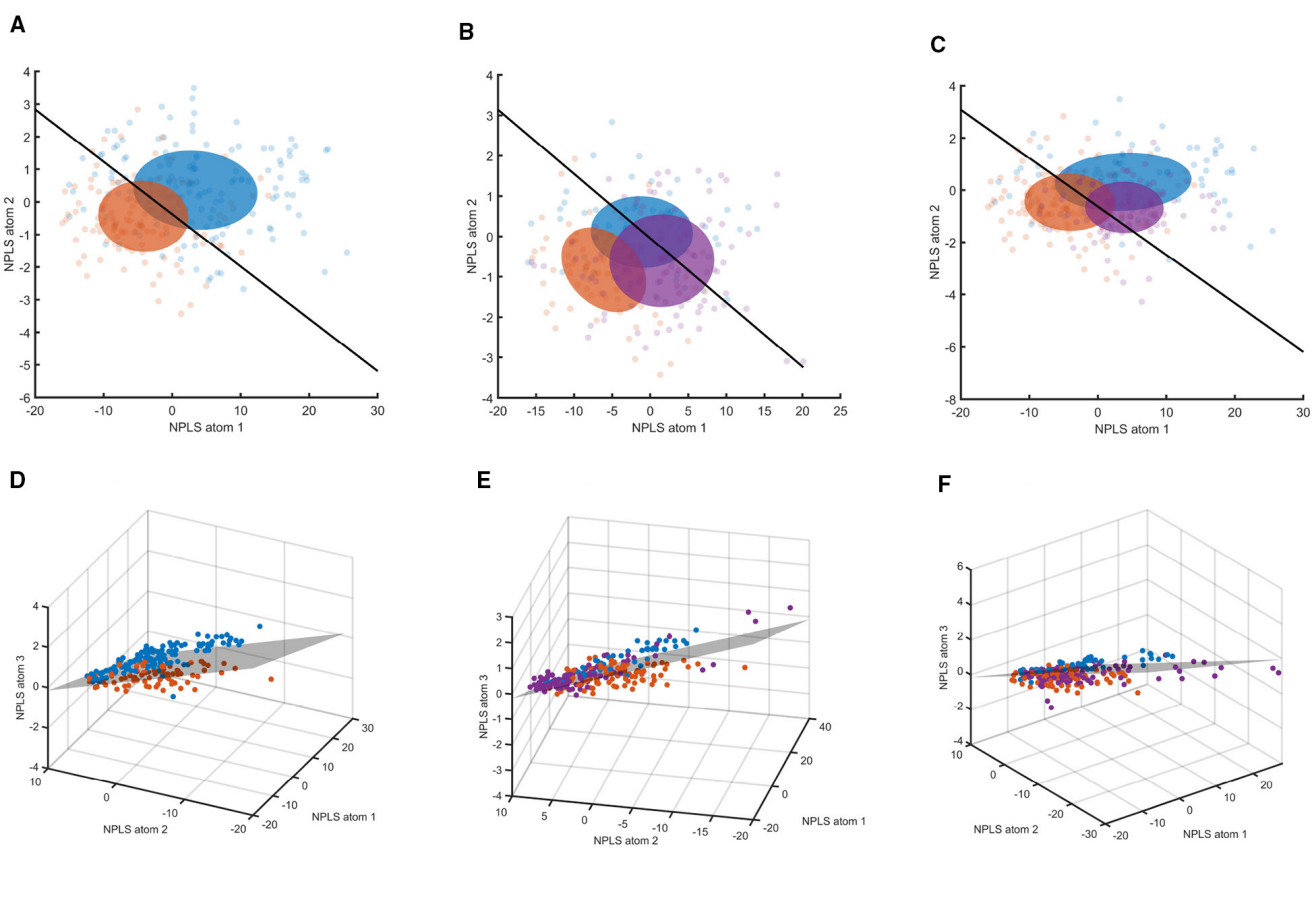
N-PLS T-scores for training and session data. The first column shows the training data, the second column shows pre-session (blue), middle virtual-reality block (purple), and post-session (red) blocks for the MI session, and the third column shows the corresponding blocks for the control session. The first row depicts Participant 05 in two latent dimensions, and the second row depicts Participant 08 in three latent dimensions. In the two-dimensional representations, ellipses are shown for each data segment to indicate the standard deviation of the data and improve visual interpretation. The black line in the two-dimensional plots represents the threshold separating low- and high-fatigue domains, while the corresponding threshold in the three-dimensional representations is shown as a gray plane. **(A)** Participant 05: training data. **(B)** Participant 05: MI session. **(C)** Participant 05: control session. **(D)** Participant 08: training data. **(E)** Participant 08: MI session. **(F)** Participant 08: control session.

The first column of Figure 12 illustrates this separation for the training data, which includes the clean pre- and post-session recordings from both the MI and control sessions. Model training was performed exclusively on clean resting-state data acquired pre- and post- both the MI and control sessions. These clean pre- and post-session resting-state recordings were used to construct the final individualized N-PLS models. Once trained, the models were kept fixed and subsequently applied to artifact-contaminated resting-state data obtained before, after, and during the VR sessions in both the MI and control conditions without any further adaptation.

The second column of Figure 12 presents the MI session data, including artifact-contaminated pre- and post-session resting-state data and the middle VR-MI block, projected into the latent space. The third column shows the corresponding control session data with the same structure. Even though this data contained artifacts, and as can be seen from Figure 12 the separation still remains sufficient.

Projecting the data from the VR session into this latent space revealed a progressive shift from the non-fatigued toward the fatigued region, consistent with the hypothesized development of fatigue. However, we also observed differences between the distributions of the pre- and post-resting-state data and those obtained during the VR sessions.

#### 3.3.2 Temporal fatigue prediction

The fatigue prediction problem was posed as a two-class classification task. For each session and for each participant, the N-PLS-LDA model generated class membership decisions along with posterior probability estimates representing the likelihood of each epoch belonging to a given class. These classification outputs were subsequently aligned with quantitative indices of signal quality, reflecting the percentage of artifacts detected within each epoch. The resulting probability scores were smoothed using a moving average with a window size of 100 epochs to reduce short-term fluctuations.

**Figure 13:**
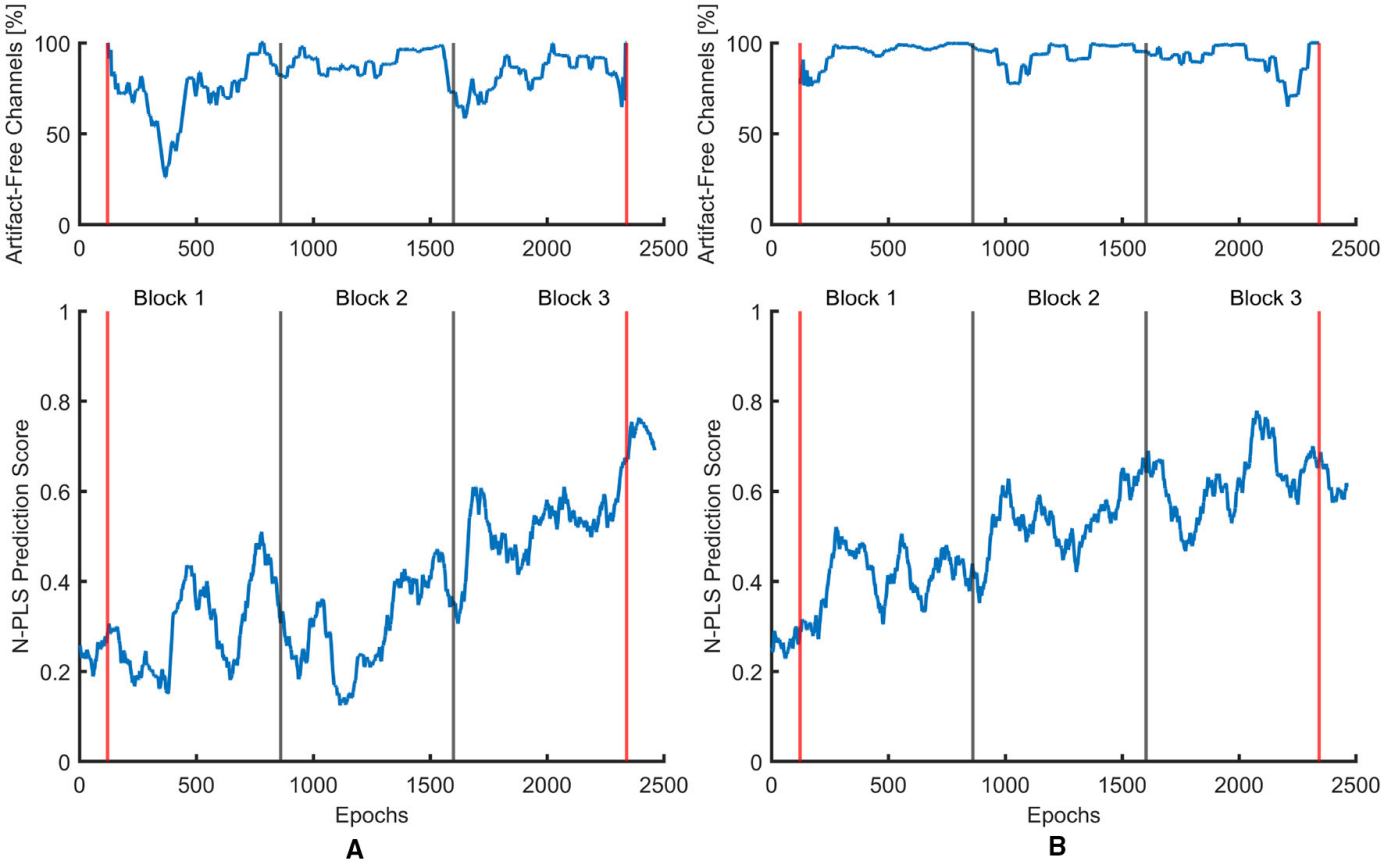
Smoothed fatigue prediction trajectories for Participant 05 using artifactual EEG data. The upper panels show the percentage of artifact-free channels per epoch, and the lower panels display the corresponding N-PLS fatigue scores. **(A)** MI session. **(B)** Control session.

Across sessions, the classifier demonstrated a consistent temporal progression, in which single-trial N-PLS predictions gradually shifted from the non-fatigued to the fatigued class. Initially, predicted points overlapped with the non-fatigued training set, but over time they moved progressively into the fatigue region. This trend was particularly evident when visualizing the model output for the artifactual (non-cleaned) data of Participant 05 in both the MI and control sessions (see Figure 13). In both conditions, the predicted fatigue probabilities exhibited a gradual increase over time, reflecting a growing likelihood of fatigue as the session progressed.

##### 3.3.2.1 Group-wise fatigue prediction

Taking our goal of achieving robust online mental fatigue estimation during rehabilitation sessions into consideration, it is crucial for the models to be resistant to artifacts. Therefore, we applied the N-PLS-LDA model to EEG data with artifacts. For all seven participants, we computed the mean N-PLS classification scores from the MI and control sessions to illustrate the temporal evolution of predicted fatigue levels throughout each session. During the VR session, each block was divided into three equal segments, which were then averaged, resulting in nine mean values of N-PLS fatigue scores.

**Figure 14:**
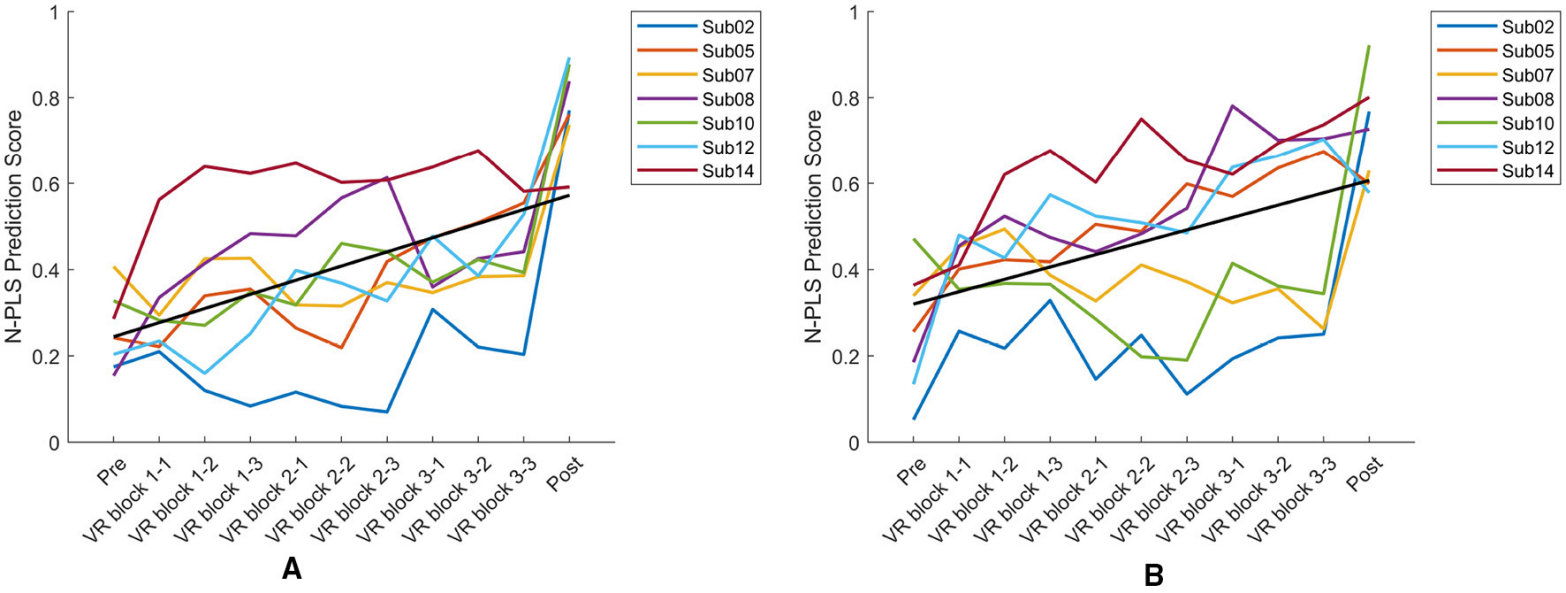
Mean N-PLS fatigue prediction trajectories for all seven participants during the left MI session **(A)** and the control session **(B)**. Predicted fatigue scores range from 0 (non-fatigued) to 1 (high fatigue), showing a progressive increase over time. The black line indicates the group-level trend across participants.

Figure 14 shows estimated mental fatigue scores across eleven points: the first corresponds to the pre-session resting-state measurement, the next nine represent predicted N-PLS fatigue scores across the VR session, and the final value reflects mental fatigue prediction at the post-session resting-state condition. For all participants, there was a noticeable increase in predicted mental fatigue scores toward the fatigued state by the end of the experiment, with occasional temporary decreases likely reflecting the brief rest periods between blocks that allowed participants to recover. This trend was evident across both experimental conditions, indicating that the N-PLS model effectively captures the gradual increase in mental fatigue over time.

As shown in Figure 14, the overall direction of change across the seven curves was assessed by fitting a linear trend line to the data. The mean curve was first obtained by averaging the corresponding data points across all seven subjects, after which linear regression was applied to this aggregated curve to characterize the global trend.

The linear fitted model is expressed as


$$\text{y}={\text{p}}_{1}\text{x}+{\text{p}}_{2},$$

where the slope p_1_ quantifies the rate and direction of change. The overall slope of the MI session was 0.0328, whereas the control session exhibited a slope of 0.0287.

### 3.4 Correlation between subjective and model-based fatigue estimates

**Figure 15:**
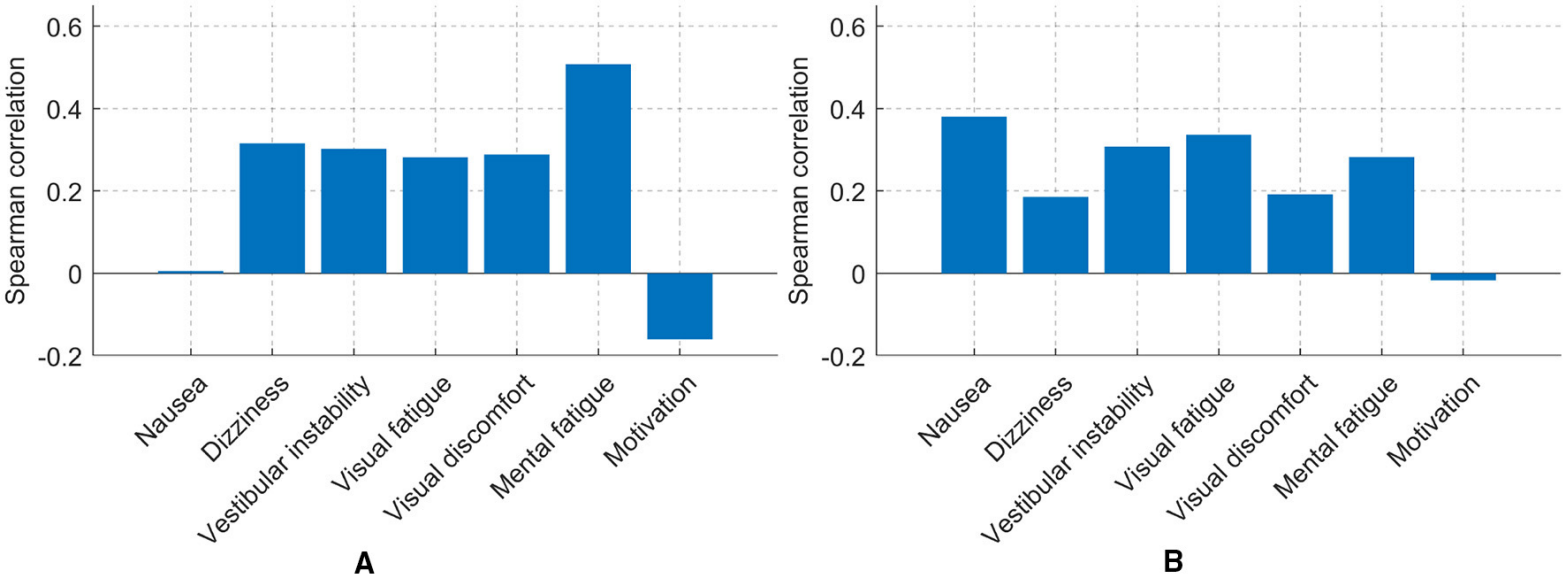
Spearman correlations between questionnaire items and N-PLS–derived fatigue estimates for the MI and control sessions. **(A)** MI session. **(B)** Control session.

Spearman correlations between individual questionnaire items and N-PLS–derived fatigue estimates revealed distinct patterns across sessions (Figure 15). In the MI session, self-reported mental fatigue showed the strongest positive correlation with predicted fatigue levels, whereas in the control session, nausea exhibited the highest association. In both sessions, motivation was negatively correlated with estimated fatigue, suggesting that higher fatigue was consistently accompanied by reduced motivation and task engagement.

## 4 Discussion

The results of this research suggest that changes in harmonic alpha band amplitude corresponded to changes in participants' mental fatigue. This relationship was supported by both subjective and objective measures, which were significantly correlated and were clearly captured by the N-PLS-LDA models. Furthermore, we were able to reliably estimate mental fatigue during the task even in the presence of artifacts, highlighting the stability of the derived fatigue-related features.

### 4.1 Subjective and objective measures

Fatigue can be assessed through objective indicators (EEG, performance, time-on-task, etc.) as well as subjective reports, and these measures sometimes diverge (Ackerman and Kanfer, 2009; Kunasegaran et al., 2023). This discrepancy reflects the distinction between physiological fatigue and the conscious feeling of fatigue: while EEG captures changes in cortical processing, subjective ratings reflect an adaptive signal prompting rest, and therefore they can follow different dynamics (Castell et al., 2011).

In our study, negative correlations between motivation and other questionnaire responses (mental fatigue, visual discomfort, etc.; see Figure 6) indicate that fatigue develops as an interplay of multiple factors rather than a single cause. This observation is consistent with Kok (2022), who highlighted the multifactorial nature of fatigue. These correlations are consistent with differences in self-reported mental states across sessions.

The MI condition required sustained attention and cognitive effort, leading to higher mental effort and cognitive fatigue, but it was also perceived as more engaging and intrinsically motivating. In contrast, the control session involved a simpler task, where participants experienced lower cognitive strain but also reduced engagement and increased monotony, promoting fatigue through under-stimulation rather than sustained effort. These observations are consistent with the underload/overload framework, which posits that mental fatigue can arise either from high cognitive demands or from insufficient stimulation (Pattyn et al., 2008). This interpretation is further supported by the correlations between self-reported measures and N-PLS based estimated fatigue levels (Figure 15), which illustrate how these different factors shape the subjective experience of fatigue.

Corresponding changes in EEG and subjective measures support this view. The self-reported increase in mental fatigue was accompanied by changes in neural activity, suggesting that participants experienced measurable fatigue during both the MI and control sessions.

Previous research has reported EEG markers of fatigue across multiple frequency bands, from delta to beta, but findings are often inconsistent. A review by Tran et al. (2020) indicates that the most robust changes are typically observed in theta and alpha activity. Alpha oscillations, in particular, are associated with top-down inhibition in frontal, central, and posterior regions during sustained attention, contributing to mental fatigue. In our study, both sessions showed an increase in alpha-band amplitude.

#### 4.1.1 Alpha oscillations and neurophysiological mechanisms

While changes in the alpha and theta bands are often reported in the literature (Tran et al., 2020; Trejo et al., 2015), the predominance of alpha effects in the present work is consistent with our paradigm, in which the predictive model was trained and applied to resting-state data.

To better understand the neural mechanisms underlying the observed fatigue, our analysis focused on alpha-band oscillations. The effect of fatigue on alpha power is one of the most consistent findings in EEG research on sustained attention and cognitive effort (Goodman et al., 2025; Boksem et al., 2005; Klimesch, 1999). Alpha oscillations are generally interpreted as indicators of functional inhibition (Klimesch, 2012), reflecting reduced cortical engagement and processing (Pfurtscheller and Lopes Da Silva, 1999). Accordingly, an increase in alpha power during fatigue signifies declining cortical excitability and attentional efficiency as mental effort continues.

Parietal alpha activity, in particular, is associated with attentional control and working memory. As fatigue accumulates, attentional resources diminish, leading to reduced top-down modulation and a shift from externally directed to internally oriented processing. EEG–fMRI studies (Laufs et al., 2003) have further linked elevated alpha power to increased activation of the default mode network (DMN), specifically in the EO condition. This system is associated with mind-wandering and self-referential thought, suggesting a transition from task-related fronto-parietal control networks toward internally focused or resting-state modes of processing. Overall, increased alpha power reflects reduced cortical activation and attentional disengagement, consistent with subjective reports of tiredness and lower motivation.

#### 4.1.2 Resting-state and VR effects

Although alpha-band activity is the predominant frequency in adult resting-state EEG, historically noted by Berger (1929), it serves an active cognitive function rather than reflecting mere idling (Klimesch, 2012), consistently manifesting as the principal spectral peak.

In our study, alpha amplitude during the VR resting-state periods was higher compared to the EO resting-state recorded both pre- and post-session (see Figure 8). EEG signals acquired in the VR environment exhibited distinct spectral profiles, which appear to reflect the influence of the immersive VR environment, the use of the HMD, and task-related engagement. A similar separation is also evident in the latent representations extracted by the N-PLS models, as shown in Figure 12. Breves and Stein (2023) found that 3D HMD-based VR induces higher cognitive load and increased cybersickness compared to 2D VR, indicating that immersive display modality can significantly increase overall cognitive demands. A similar effect may be present in this study when comparing non-VR resting-state conditions with 3D immersive VR, suggesting that the observed differences could partly reflect differences in baseline cognitive load induced by the HMD environment.

One explanation for these observations is based on the inhibitory role of alpha oscillations. Alpha activity actively participates in attention, information processing, and controlled access to memory (Klimesch, 2012). In this context, higher alpha amplitude corresponds to stronger inhibition, defined as the active suppression of task-irrelevant cortical regions. Increased alpha power therefore reduces neural excitability, limits interference, and facilitates selective attention and controlled access to relevant information. These results suggest that the VR-HMD environment modulates cortical excitability and reorganizes resting-state dynamics, with elevated alpha activity potentially representing compensatory inhibition to manage heightened sensory and attentional demands. This suggests that VR-based resting conditions may involve a higher baseline cognitive load than conventional EO or EC resting-state recordings due to continuous sensory and attentional processing demands.

### 4.2 Mental fatigue estimation

The mental fatigue classifier achieved an average accuracy of 83.49 ± 6.34%, estimated from pre- and post-session resting-state data using 5-fold cross-validation. The model performs predictions on short 2-s EEG epochs, meaning that each classification is based on a very limited temporal window of neural activity. Under these conditions, some variability in accuracy across participants is expected, as the onset and progression of mental fatigue differ substantially between individuals and are reflected in subject-specific EEG dynamics. Consequently, the reported performance demonstrates that reliable fatigue estimation can be achieved even at a fine temporal resolution suitable for continuous monitoring during BCI-HMD rehabilitation tasks.

N-PLS–estimated fatigue showed similar trends across MI and control sessions (Figure 14) Both sessions exhibited positive slopes, indicating an overall increase in predicted fatigue over time. The slightly larger slope observed in the MI condition suggests a somewhat steeper increase in fatigue compared with the control condition. Correlations with self-reported measures (Figure 15) indicate that mental effort, motivation, and discomfort tracked the model's predictions, aligning subjective experience with EEG markers.

We applied N-PLS models to artifactual EEG data to estimate mental fatigue scores. The results indicate that the models remain stable under these conditions, producing consistent fatigue estimates despite the presence of artifacts in the input data, suggesting that the estimated scores are primarily driven by meaningful EEG activity.

Our results confirm that modeling mental fatigue benefits greatly from an individualized approach that accounts for each participant's EEG spectral profile and its temporal evolution. The initial modeling further underscored the necessity of critically evaluating the extracted components. Early iterations revealed a prominent μ component, which was not aligned with the objectives of fatigue detection. While the MI task is central to the rehabilitation objectives, the closed-loop BCI-HMD setup aims to optimize post-stroke therapy through adaptive MI tasks. In this context, it is essential that the fatigue model captures genuine fatigue-related neural changes rather than task-driven sensorimotor activity. To address this, the μ component was removed prior to model estimation. Following this correction, the alpha band emerged as the primary frequency band associated with fatigue.

Furthermore, the findings suggest that a generalized EEG-based fatigue model, such as the one applied across both MI and control sessions in this study, may be transferable across days for a given participant, provided that appropriate recentering or threshold adjustment is implemented.

The N-PLS algorithm extracts component weights iteratively, each time maximizing the covariance with the remaining unexplained portion of the dependent variable (Bro, 1996). The first N-PLS component captures the largest share of fatigue-related variance and, in our case, was dominated by parieto-occipital alpha activity. As discussed in Section 4.1.2, such posterior alpha modulation is frequently reported in the literature. These patterns can be interpreted through several established mechanisms of mental fatigue (Salihu et al., 2022).

Resource depletion theory (Helton and Warm, 2008) links sustained cognitive effort to reduced resources in the cognitive control network, which leads to a decline in performance and manifests as posterior alpha increases, indicating mental fatigue. As explained in a review on the neural mechanisms underlying mental fatigue (Salihu et al., 2022), this theory is closely related to the alternative “waste disposal” hypothesis. This hypothesis suggests that mental fatigue does not develop due to depleted cognitive resources, but rather because prolonged task engagement leads to the accumulation of neurotoxic metabolic byproducts, resulting in decreased activity in control regions. Increased alpha activity may reflect compensatory inhibition. Temporary reductions in estimated fatigue between blocks may therefore represent partial recovery through the clearance of accumulated waste products.

Overall, the N-PLS model captures shared neural mechanisms underlying subjective fatigue, linking parieto-occipital alpha modulation to both resource use and neuroprotective regulation across tasks.

### 4.3 Limitations

Several limitations of this study should be acknowledged. The proposed modeling approach is subject-specific, reflecting the well-established variability of EEG spatial–spectral patterns across individuals. While this limits direct generalization to population-level models, it aligns with standard practice in brain–computer interface applications, where individualized calibration is typically required.

Second, subjective fatigue was assessed using a custom-designed questionnaire, which was not a validated psychometric instrument. Some items combined multiple symptoms (e.g., visual strain and drowsiness), which may introduce ambiguity and reduce specificity of the measured constructs. Therefore, subjective measures should be interpreted as supportive rather than definitive, and future studies should incorporate validated fatigue and cybersickness scales or refine item design to ensure independence of measured dimensions.

Third, exclusion criteria and pre-session conditions were based on self-reported compliance, without formal clinical screening or objective verification. While this approach reflects typical experimental practice, it may introduce uncontrolled variability related to participants' physiological or cognitive state.

Finally, although the experimental setup was designed to approximate realistic BCI-HMD conditions, the study was conducted in a controlled laboratory environment with healthy participants, and therefore may not fully capture the complexity of real-world clinical neurorehabilitation settings.

## 5 Conclusion

This study demonstrates that changes in alpha-band EEG activity provide a reliable indicator of mental fatigue during both MI and control tasks performed in a VR-based HMD environment. The results show that fatigue in both conditions engages overlapping neural mechanisms, most prominently reflected in parieto-occipital alpha modulation. Across participants, subjective reports, behavioral responses, and N-PLS–derived EEG features converged toward a coherent model of fatigue as a gradual withdrawal of attentional control and cortical engagement, consistent with established neurophysiological accounts of functional inhibition during sustained effort.

The consistency of fatigue estimation across conditions suggests that individualized models can generalize across days when appropriately recentered. Additionally, the clear distinctions between VR and non-VR resting-state EEG highlight the importance of acquiring baseline measurements directly within the immersive environment to ensure that the reference state reflects the user's actual sensory and attentional demands.

Future work should focus on refining the model and examining the stability of these fatigue-related markers in clinical populations, particularly post-stroke patients. The integration of real-time fatigue monitoring into adaptive BCI-HMD systems represents a promising step toward improving usability, optimizing task difficulty, and enhancing the overall effectiveness of VR-supported motor neurorehabilitation.

## Data Availability

The raw data supporting the conclusions of this article will be made available by the authors, without undue reservation.
